# Validation of the AJCC prognostic stage for HER2-positive breast cancer in the ShortHER trial

**DOI:** 10.1186/s12916-019-1445-z

**Published:** 2019-11-21

**Authors:** Maria Vittoria Dieci, Giancarlo Bisagni, Alba A. Brandes, Antonio Frassoldati, Luigi Cavanna, Francesco Giotta, Michele Aieta, Vittorio Gebbia, Antonino Musolino, Ornella Garrone, Michela Donadio, Anita Rimanti, Alessandra Beano, Claudio Zamagni, Hector Soto Parra, Federico Piacentini, Saverio Danese, Antonella Ferro, Katia Cagossi, Samanta Sarti, Anna Rita Gambaro, Sante Romito, Viviana Bazan, Laura Amaducci, Gabriella Moretti, Maria Pia Foschini, Sara Balduzzi, Roberto Vicini, Roberto D’Amico, Gaia Griguolo, Valentina Guarneri, Pier Franco Conte

**Affiliations:** 1 Department of Surgery, Oncology and Gastroenterology, University of Padova, Padova, Italy; 2 Medical Oncology 2, Istituto Oncologico Veneto - IRCCS, Via Gattamelata 64, 35128 Padova, Italy; 3 Department of Oncology and Advanced Technologies, Oncology Unit, Azienda USL-IRCCS, Reggio Emilia, Italy; 4 Medical Oncology, Azienda Unità Sanitaria Locale di Bologna-IRCCS Istituto delle Scienze Neurologiche, Bologna, Italy; 5 Clinical Oncology, Department of Morphology, Surgery and Experimental Medicine, S Anna University Hospital, Ferrara, Italy; 6 Department of Oncology-Hematology, G. da Saliceto Hospital, Piacenza, Italy; 7 IRCCS Istituto Tumori “Giovanni Paolo II” di Bari, Bari, Italy; 8 Division of Medical Oncology, IRCCS-CROB, Referral Cancer Center of Basilicata, Rionero Vulture, Italy; 9 Medical Oncology, Casa di Cura La Maddalena, University of Palermo, Palermo, Italy; 10 Medical Oncology Unit, University Hospital of Parma, Parma, Italy; 11 Medical Oncology, A.O. S. Croce and Carle Teaching Hospital, Cuneo, Italy; 12 Department of Medical Oncology 1, Città della Salute e della Scienza Hospital, Turin, Italy; 13 Medical Oncology, Azienda Ospedaliera di Mantova, Mantova, Italy; 14 Policlinico S.Orsola-Malpighi, SSD Oncologia Medica Addarii, Bologna, Italy; 15 Medical Oncology Unit, AOU Policlinico Vittorio Emanuele, Catania, Italy; 16 Division of Medical Oncology Department of Medical and Surgical Sciences for Children & Adults, University Hospital of Modena, Modena, Italy; 17 Department of Gynecology and Obstetrics, Ospedale S. Anna, Turin, Italy; 18 Rete clinica senologica - Oncologia medica S. Chiara, Trento, Italy; 19 Breast Unit Ausl Modena, Ramazzini Hospital, Carpi, Italy; 20 Istituto Scientifico Romagnolo per lo Studio e la Cura dei Tumori (IRST) IRCCS, Meldola, Italy; 21 Oncology Unit, Luigi Sacco Hospital, Milan, Italy; 22 Medical Oncology, A.O.U. “Ospedali Riuniti”, Foggia, Italy; 23 Department of Biomedicine, Neurosciences and Advanced Diagnostics, University of Palermo, Palermo, Italy; 24 Medical Oncology Unit, Ospedale degli Infermi Faenza, Faenza, Italy; 25 Department of Biomedical and Neuromotor Sciences, University of Bologna, Unit of Anatomic Pathology at Bellaria Hospital, Bologna, Italy; 26 Department of Medical and Surgical Sciences for Children & Adults, University of Modena, Modena, Italy; 27 Azienda Ospedaliero-Universitaria di Modena, Modena, Italy

**Keywords:** HER2-positive, Breast cancer, Trastuzumab, Prognostic stage, 8th AJCC

## Abstract

**Background:**

The 8th edition of the American Joint Committee on Cancer (AJCC) staging has introduced prognostic stage based on anatomic stage combined with biologic factors. We aimed to validate the prognostic stage in HER2-positive breast cancer patients enrolled in the ShortHER trial.

**Methods:**

The ShortHER trial randomized 1253 HER2-positive patients to 9 weeks or 1 year of adjuvant trastuzumab combined with chemotherapy. Patients were classified according to the anatomic and the prognostic stage. Distant disease-free survival (DDFS) was calculated from randomization to distant relapse or death.

**Results:**

A total of 1244 patients were included. Compared to anatomic stage, the prognostic stage downstaged 41.6% (*n* = 517) of patients to a more favorable stage category.

Five-year DDFS based on anatomic stage was as follows: IA 96.6%, IB 94.1%, IIA 92.4%, IIB 87.3%, IIIA 81.3%, IIIC 70.5% (*P* < 0.001). Five-year DDFS according to prognostic stage was as follows: IA 95.7%, IB 91.4%, IIA 86.9%, IIB 85.0%, IIIA 77.6%, IIIC 67.7% (*P* < 0.001). The C index was similar (0.69209 and 0.69249, *P* = 0.975).

Within anatomic stage I, the outcome was similar for patients treated with 9 weeks or 1 year trastuzumab (5-year DDFS 96.2% and 96.6%, *P* = 0.856). Within prognostic stage I, the outcome was numerically worse for patients treated with 9 weeks trastuzumab (5-year DDFS 93.7% and 96.3%, *P* = 0.080).

**Conclusions:**

The prognostic stage downstaged 41.6% of patients, while maintaining a similar prognostic performance as the anatomic stage. The prognostic stage is valuable in counseling patients and may serve as reference for a clinical trial design. Our data do not support prognostic stage as guidance to de-escalate treatment.

**Trial registration:**

EUDRACT number: 2007-004326-25; NCI ClinicalTrials.gov number: NCT00629278.

## Introduction

Advances in medical treatment have significantly improved the prognosis of human epidermal growth factor 2 (HER2)-positive early breast cancer (BC) patients over time and led to establish chemotherapy combined with 1 year of trastuzumab as the standard adjuvant treatment [1].

The impact of prognostic/predictive biomarkers on the outcome of patients treated with appropriate standard systemic treatment has been considered by the American Joint Committee on Cancer (AJCC) Staging System panel in the update of the breast cancer staging. Based on the incorporation of biologic factors (histologic grade, estrogen receptor, progesterone receptor, HER2, and multigene panels) to the classic anatomic stage, the 8th edition of the AJCC breast cancer staging system has introduced prognostic stage, which was developed using data from patients identified in the National Cancer Database (2010–2011) and then validated in large cohorts of patients from the MD Anderson Cancer Center and the California Cancer Registry [2–7]. These studies allowed to confirm the improved prognostic performance of the prognostic stage as compared to the anatomic stage in the general breast cancer patients’ population. The most recently updated version of the prognostic stage was released after the results of the validation study highlighted that a proportion of patients could not be assigned a specific prognostic stage [7]. Therefore, the prognostic staging system was refined to include all the possible combinations of anatomic stages and biomarkers [8]. As declared by the AJCC staging panel, the actual prognostic stage will undergo frequent updates, based on future validation studies in large databases of patients treated with state-of-the-art therapies [4, 6]. Several studies, all conducted in retrospective patient cohorts, have been reported in the last couple of years, overall corroborating the prognostic stage as a more accurate discriminator of breast cancer patients’ outcome as compared to the anatomic stage. However, it has to be pointed out that many of these studies used data from the National Cancer Database or the SEER (Surveillance, Epidemiology, and End Results) registry covering a period of time including years 2010 and 2011. Considering the overlap between the National Cancer Database and the SEER, these studies included data that were previously used by the AJCC panel to develop the prognostic score. Moreover, most of these studies, including the main validation studies by the AJCC panel, did not report detailed analysis of distinct breast cancer subtypes, with no study specifically focused on HER2-positive disease. Furthermore, even in the most robust cohorts, exposure to trastuzumab was not reported or not homogeneous among HER2-positive patients (literature review in Additional file 1) [7, 9–23]. This aspect is a relevant caveat, since the assumption at the basis of the adoption of the prognostic stage is that patients are offered adequate systemic treatment based on biologic characterization [2, 4, 6].

One of the major clinical needs for HER2-postive BC patients is an accurate risk stratification to guide escalated and de-escalated strategies to ensure the most effective treatment along with a more rationale resource allocation [24]. One of the most important goals of staging is to help clinicians define a treatment plan [5]; therefore, the evaluation of outcome prediction by the prognostic stage in HER2-positive patients cohorts treated with standard therapy is a key step in order to define its potential role as tool to guide de-escalated therapeutic choices. For this kind of investigation, a randomized trial testing de-escalated against standard treatment represents the ideal setting.

In this study, we aimed to validate the prognostic stage in HER2-positive BC patients treated with adjuvant chemotherapy combined with 1 year or 9 weeks trastuzumab in the randomized ShortHER trial [25].

## Methods

### Patients

The ShortHER trial (NCT00629278) is a phase 3 trial of adjuvant therapy that randomized 1253 patients with HER2-positive early BC to anthracycline and taxane-based chemotherapy combined with 1 year (long) or 9 weeks (short) trastuzumab. Study characteristics and results are reported elsewhere [25].

### Staging

In the present analysis, patients were classified according to the anatomic stage, based on tumor size (T) and nodal status (N), and to the prognostic stage that takes into account T, N, estrogen receptor, progesterone receptor, histologic grade, and HER2 status. The most recent version of the 8th AJCC edition was used as reference [8]. Histologic grade, hormone receptor expression, and HER2 status were based on local pathology. According to the AJCC staging manual, for the present analysis, estrogen receptor and progesterone receptor expression was classified as positive in case of staining in > 1% of tumor cells.

Once the anatomic and prognostic stages were applied, patients with discordant stage assignment were defined as follows:

- Those patients moved to a more favorable stage category with the prognostic stage as compared to the anatomic stage were defined as downstaged;
- Those patients moved to a less favorable stage category with the prognostic stage as compared to the anatomic stage were defined as upstaged.

### Statistical analysis

Statistical analyses were performed using IBM SPSS v.24 and R project for Statistical Computing [26]. Distant disease-free survival (DDFS) was calculated from randomization until relapse at a distant site or death, whichever first.

Kaplan-Meier method was used to estimate survival curves. The log-rank test was used to compare stage categories. The Harrel concordance index (C index) was calculated for each of the two staging systems. Difference between the C index of the anatomic and prognostic stage models was tested by using “compareC” package in R [26]. Cox proportional regression models were used to calculate hazard ratios (HRs) and 95% confidence intervals (CIs). The log-rank test *χ*^2^ statistic, and its *P* value were used to explore the discrimination between groups. The significance level was *P* < 0.05. All tests were two-sided.

## Results

### Stage classification

Complete data for classification according to the anatomic and the prognostic stage were available for 1244 patients. Patients’ characteristics are reported in Table 1. The comparison of anatomic and prognostic stage classifications is summarized in Table 2.

**Table 1:** Patients’ characteristics

Characteristics	*N* (%)
Age, years	
< 60	795 (69.3)
> 60	449 (36.1)
Histologic grade	
1	8 (0.7)
2	363 (29.6)
3	857 (69.8)
Menopausal status	
Premenopause	446 (35.9)
Postmenopause	797 (64.1)
Estrogen receptor (> 1% cut-off)	
Negative	354 (28.5)
Positive	890 (71.5)
Progesterone receptor (> 1% cut-off)	
Negative	495 (39.8)
Positive	749 (60.2)
Pathologic T stage	
pT1	763 (61.4)
pT2	450 (36.2)
pT3	28 (2.3)
pT4	1 (0.1)
Pathologic lymph nodes stage	
pN0	672 (54.0)
pN1mic	69 (5.5)
pN1	321 (25.8)
pN2	116 (9.3)
pN3	66 (5.3)
Arm	
A Long	624 (50.2)
B Short	620 (49.8)

*Abbreviation*: *N* number

**Table 2:** Comparison of anatomic and prognostic stage classifications in patients enrolled in the ShortHER trial

	AJCC prognostic stage						
	IA	IB	IIA	IIB	IIIA	IIIB	Tot
	*N* (%)	*N* (%)	*N* (%)	*N* (%)	*N* (%)	*N* (%)	*N* (%)
AJCC anatomic stage							
IA	469 (100)	0	0	0	0	0	469 (37.7)
IB	40 (100)	0	0	0	0	0	40 (3.2)
IIA	222 (55.6)	24 (6.0)	153 (38.3)	0	0	0	399 (32.1)
IIB	2 (1.3)	92 (61.7)	0	55 (36.9)	0	0	149 (12.0)
IIIA	0	23 (19.0)	48 (39.7)	0	50 (41.3)	0	121 (9.7)
IIIB	0	0	0	0	0	0	0
IIIC	0	0	0	0	9 (13.6)	57 (86.4)	66 (5.3)
Tot	733 (58.9)	139 (11.2)	201 (16.2)	55 (4.4)	59 (4.7)	57 (4.6)	1244 (100)

*Abbreviations*: *AJCC* American Joint Committee on Cancer, *N* number

The rate of concordance was 58.4% (*n* = 727 patients), whereas 517 patients (41.6%) had a discordant stage category assignment. All discordant cases were downstaged by the prognostic stage:

- 100% of anatomic stage IB patients (*n* = 40) were re-classified as IA
- 61.6% of anatomic stage IIA patients (*n* = 246) were re-classified as IB (6.0%) or IA (55.6%);
- 63.0% of anatomic stage IIB patients (*n* = 94) were re-classified as IA (1.3%) or IB (81.7%);
- 58.7% of anatomic stage IIIA patients were re-classified as IB (19.0%) or IIA (39.7%);
- 100% of anatomic stage IIIC patients (*n* = 66) were re-classified as IIIA (13.6%) or IIIB (86.4%).

Among downstaged patients, the change was by one stage down for 23.4% (*n* = 121), by two stages down for 71.8% (*n* = 371), and by three stages down for 4.8% (*n* = 25) of cases.

### Survival analysis

**Fig. 1:**
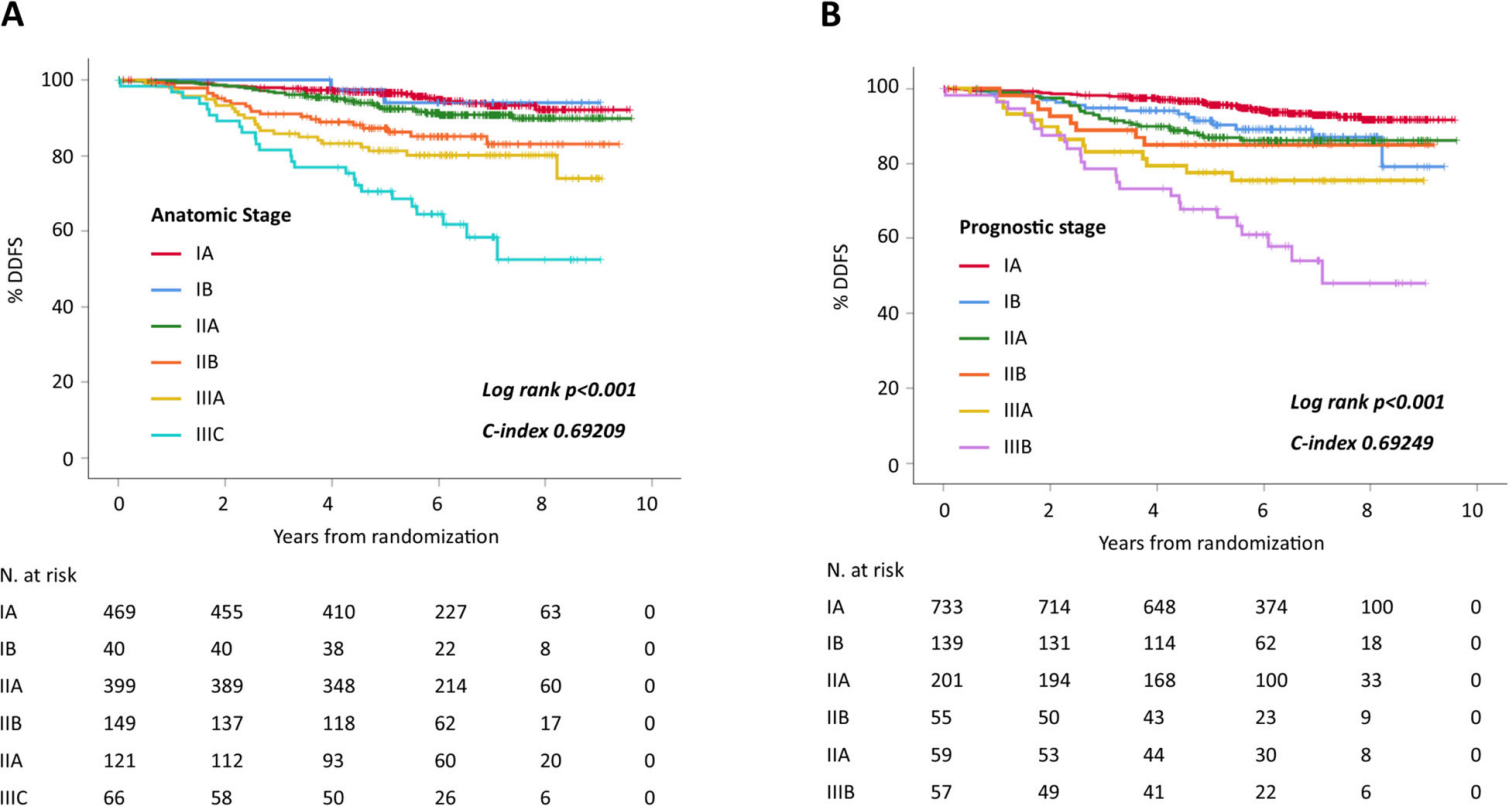
Kaplan-Meier DDFS curves by anatomic stage (**a**) and prognostic stage (**b**)

Median follow-up was 6.1 years. Five-year DDFS rates and their 95% confidence interval for stage categories according to the anatomic and prognostic stage classifications are reported in Table 3, survival curves are shown in Fig. 1.

**Table 3:** Five-years DDFS rates by stage category according to the anatomic and prognostic stage classifications

Stage	Anatomic stage		Prognostic stage	
	*N* (%)	5-yr DDFS % (95% CI)	*N* (%)	5-yr DDFS % (95% CI)
IA	469 (37.7)	96.6 (95.0–98.3)	733 (58.9)	95.7 (94.2–97.3)
IB	40 (3.2)	94.1 (86.4–100)	139 (11.2)	91.4 (86.6–96.4)
IIA	399 (32.1)	92.4 (89.7–95.2)	201 (16.2)	86.9 (82.2–91.9)
IIB	149 (12.0)	87.3 (82.0–93.0)	55 (4.4)	85.0 (76.0–95.2)
IIIA	121 (9.7)	81.3 (74.5–88.7)	59 (4.7)	77.6 (67.6–89.1)
IIIB	–	–	57 (4.6)	67.7 (56.5–81.2)
IIIC	66 (5.3)	70.5 (50.2–82.6)	–	–

*Abbreviations*: *N* number, *yr* year, *DDFS* distant disease-free survival

Both models showed the ability to stratify patients at different outcome (log-rank *P* < 0.001). The C index was 0.69209 for the anatomic stage and 0.69249 for the prognostic stage, with no significant difference (*P* = 0.975). With prognostic stage, 58.9% of patients were classified as stage IA and showed excellent outcome after adjuvant chemotherapy and trastuzumab (5-year DDFS 95.7%, 95%CI 94.2–97.3%). However, within each of the stage categories, the outcome was numerically inferior for the prognostic stage groups (Table 3).

Table 4 shows Cox regression analysis for DDFS according to anatomic and prognostic stage, with stage IA as reference category. With anatomic stage, the prognosis of stage IB and IIA patients was not statistically different as compared to stage IA patients. With prognostic stage, all stage categories showed significantly worse outcome as compared to stage IA. We further explored the stage discrimination by focusing on stages I–II and looking at the log-rank *χ*^2^ and its *P* value in paired comparisons. For IB vs IA, the *χ*^2^ statistics was 0.014 (*P* = 0.906) for anatomic stage and 5.930 (*P* = 0.015) for prognostic stage. For IIA vs IB, the *χ*^2^ statistics was 0.579 (*P* = 0.447) for anatomic stage and 0.263 (*P* = 0.608) for prognostic stage. For IIB vs IIA, the *χ*^2^ statistics was 5.322 (*P* = 0.0.021) for anatomic stage and 0.165 (*P* = 0.686) for prognostic stage. A higher *χ*^2^ statistic indicates a higher group separation. The results of the Cox regression analysis and those of the paired log-rank tests indicate that, for patients with stage I–II disease, the largest prognostic discrimination is between stage IIB and previous stages for anatomic stage and between stage IB and IA for prognostic stage.

**Table 4:** Cox regression DDFS analysis

	Anatomic stage		Prognostic stage	
	HR (95%CI)	*P*	HR (95%CI)	*P*
IA	Ref		Ref	
IB	0.93 (0.22–3.92)	0.917	2.02 (1.12–3.64)	0.020
IIA	1.60 (0.95–2.71)	0.079	2.36 (1.45–3.85)	0.001
IIB	3.02 (1.68–5.42)	< 0.001	2.78 (1.31–5.93)	0.008
IIIA	4.14 (2.35–7.29)	< 0.001	4.50 (2.46–8.24)	< 0.001
IIIB	–	–	8.8 (5.33–14.54)	< 0.001
IIIC	8.58 (4.90–15.02)	< 0.001	–	–

*Abbreviations*: *HR* hazard ratio, *CI* confidence interval, *P P* value

### Short vs long trastuzumab in stage I patients

**Fig. 2:**
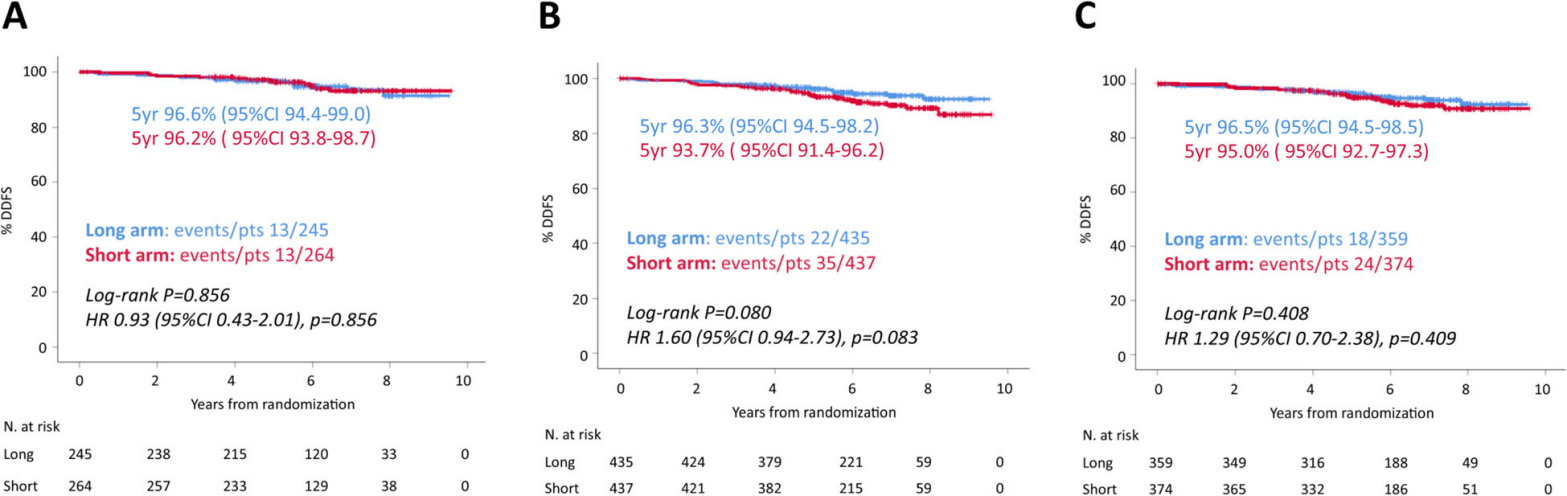
Kaplan-Meier DDFS curves for patients treated in the short (9 weeks trastuzumab) vs the long (1 year trastuzumab) arm according to stage categories: anatomic stage I patients (**a**), prognostic stage I patients (**b**), prognostic stage IA patients (**c**)

Analyses comparing DDFS of stage I patients treated with 9 weeks vs 1 year trastuzumab were conducted (Fig. 2). The outcome of anatomic stage I patients (*n* = 509) was excellent irrespectively of trastuzumab duration (5-year DDFS 96.2%, 95%CI 93.8–98.7% in the short arm and 96.6%, 95%CI 94.4–99.0% in the long arm). Among prognostic stage I patients (*n* = 872), those who received 9 weeks trastuzumab had a non-significant numerically inferior DDFS (5-year DDFS 93.7%, 95%CI 91.4–96.2% vs 96.3%, 95%CI 94.5–98.2%, log-rank *P* = 0.080; HR 1.60 95%CI 0.94–2.73, *P* = 0.083). When limiting the analysis to patients with prognostic stage IA, the absolute difference in 5-year DDFS was reduced to 1.5% (95.0%, 95%CI 92.7–97.3% in the short arm vs 96.5%, 95%CI 94.5–98.5% in the long arm, log-rank *P* = 0.408; HR 1.29, 95%CI 0.70–2.38, *P* = 0.409).

## Discussion

This is the first study (i) evaluating the performance of prognostic AJCC stage specifically for early HER2-positive BC patients treated with adjuvant chemotherapy and trastuzumab, (ii) evaluating the performance of prognostic AJCC in a prospective randomized trial, and (iii) validating the prognostic AJCC in a European patients’ cohort. Our findings show a similar prognostic performance for prognostic and anatomic stage, despite prognostic stage reallocated a substantial proportion of patients (41.6%) to a more favorable stage category. Previous studies in general BC patient populations have described a reallocation rate with prognostic stage most frequently reported around 40–60% (range 18–74%; Additional file 1) [7, 9–23]. In the California Cancer Registry, including 54,727 patients in anatomic stages I to IV, 31.0% and 20.6% of patients were assigned to a more favorable and less favorable stage category with prognostic stage, respectively [7]. Only a few studies reported the discrepancy between the two stage models specifically for HER2-positive BC patients, leading to non-univocal results (Additional file 1) [9, 14, 21, 23]. A large cohort from the National Cancer Database including *n* = 60,155 HER2-positive BC patients showed 29.4% and 0% rates of downstaging and upstaging, respectively [9]. Another study showed that 35.8% and 40.7% of HER2-positive patients (*n* = 1982) were classified as stage I by anatomic stage and prognostic stage, respectively [15]. The rate of downstaging (58.4%) was higher in our study, and consequently, the enrichment in stage I patients with prognostic stage was also more evident. The high prevalence of hormone receptor-positive patients in the ShortHER population (68%) might have contributed to substantial downstaging. It should be highlighted that the ShortHER population reflects the characteristics of HER2-positive patients commonly treated in contemporary clinical practice [25, 27].

Our data show that the substantial downstaging of patients with the prognostic stage did not affect the performance of the model which was maintained similar to anatomic stage (*P* = 0.975 for C index comparison). In this context, available literature data focused on HER2-positive patients are scanty. Moreover, their interpretation is extremely limited by the lack of homogenous treatment or lack of information about it (Additional file 1) [18, 23]. The largest cohort of HER2-positive patients analyzed for survival outcome according to prognostic stage included 562 cases (mostly not treated with trastuzumab) and showed a good 10-year disease-specific survival for prognostic stage I patients (> 96%), but did not report overall model performance [18].

As previously discussed, prognostic stage led to an enrichment in stage I (70% vs 40.9% anatomic stage) and more specifically in stage IA patients (58.9% vs 37.7%). The pairwise comparisons conducted in stage I–IIA patients suggest that the prognostic stage better discriminated the group of patients with the best prognosis among others (IA), whereas with anatomic stage there was no significant difference in outcome among patients in stages IA, IB, and IIA. However, when looking at absolute survival rates, stage IA patients had slightly numerically inferior outcome as compared to stage IA groups by anatomical stage. The numerically worse outcome for prognostic stage and matched anatomic stage categories was evident for all stage groups. In synthesis, the prognostic stage, by recognizing the prognostic effect of biomarkers, results in a shift from a worse to a better stage category (mostly to stage IA or stage IB) of a large number of patients as compared to the anatomic stage. One of the consequences of this shift is a better separation of stage groups in terms of DDFS, especially in stage I–IIA patients. However, intuitively, in absolute terms, the outcome of prognostic stage IA and IB patients, being enriched by patients with a worse anatomic stage category, is somehow diluted and results numerically inferior to the corresponding anatomic stage. Moreover, prognostic stage > IIA categories are depleted vs the same anatomic stage category in patients with a better prognosis; again, as a consequence, the outcome of prognostic stage > IIA groups is numerically inferior to the corresponding anatomic stage. The main implication is that the prognostic stage is more valuable as anatomic stage as a tool to counsel patients about their prognosis: by applying the prognostic stage, more patients would be regrouped in more favorable stage categories and would be informed about a good outcome as compared to the anatomic stage. To the other side, the prognostic stage identifies a more restricted number of patients with far poorer outcomes. However, what clinicians have to keep in mind when counseling patients is that in absolute terms the estimated outcome for a given prognostic stage category might not correspond to the estimation for the same anatomic category.

An appropriate identification of patients at excellent outcome with standard adjuvant treatment is key to identifying those patients who may be offered de-escalated treatment strategies. Treatment de-escalation for HER2-positive patients with anthracycline-free regimens as the paclitaxel-trastuzumab schedule is already administered in clinical practice based on anatomic stage, mostly for patients with stage I disease [28, 29]. We explored whether prognostic stage I may be of value in identifying patients for de-escalated therapies. Our results suggest that if anatomic stage I seems a good parameter to guide de-escalated therapeutic choices, this may not be the case for prognostic stage I. Indeed, prognostic stage I patients treated with short trastuzumab had an absolute 3% worse DDFS rate at 5 years as compared to patients enrolled in the long trastuzumab arm. When restricting the analysis to prognostic stage IA, there was still an absolute 1.5% difference in 5-year DDFS favoring the long arm. However, this result was not statistically significant and was based on a difference of just six events between the two arms. Although these were exploratory, unplanned, and unpowered analyses that should be interpreted with caution, the results can be considered as hypothesis-generating that require further testing in similar trials. To note, the acceptable absolute difference in outcome to consider a de-escalated treatment as safe is currently debated [30]. If our results will be confirmed in further studies, the two staging systems will be recognized as providing divergent information in the context of patient selection for treatment de-escalation, possibly posing a challenge in the implementation of the prognostic stage in clinical practice.

Our study has strengths: this is the first study evaluating the prognostic performance of prognostic stage in a cohort of HER2-positive patients, all receiving chemotherapy and trastuzumab; patient population is derived from a prospective trial; 99% of patients had sufficient data for the present analysis; the study design allowed to explore short vs long trastuzumab in stage-defined groups.

Main limitations of this study include the choice of the survival endpoint (DDFS) which is different from the one used to develop and validate the prognostic stage (BC-specific survival) [7]. In the ShortHER trial, actual median follow-up does not allow for a mature evaluation of BC-specific survival in this population of patients. Therefore, we opted to use DDFS as a surrogate of BC-specific survival considering the lethal nature of DDFS events. Another limitation is the reduced sample size in stage-defined groups, limiting the power of direct comparisons.

## Conclusions

In conclusion, the AJCC prognostic stage is valuable in counseling patients regarding their prognosis and may serve as reference for clinical trial design and sample size estimation. Our data do not support the assumption that prognostic stage may also guide treatment de-escalation, thus more information from other randomized trials are needed. These findings fill the present void of appraising the clinical validity and utility of prognostic staging in HER2-positive patients. Research into integrated models of risk stratification tailored at fulfilling the need for clinically useful tools to guide de-escalated therapeutic choices is highly encouraged.

## Supplementary information

**Additional file 1.** Summary of details of studies aimed at evaluating the American Joint Committee on Cancer (AJCC) 8th edition breast cancer prognostic staging.

## Data Availability

The datasets used and/or analyzed during the current study are available from the corresponding author on reasonable request.
