# Supplementary material for: Validation of the AJCC prognostic stage for HER2-positive breast cancer in the ShortHER trial
Source: BMC Med. 2019 Nov 21;17:207. doi: 10.1186/s12916-019-1445-z (PMC6868696; doi:10.1186/s12916-019-1445-z)
Supplement: Supplementary file 1 — Additional file 1. Summary of details of studies aimed at evaluating the American Joint Committee on Cancer (AJCC) 8th edition breast cancer prognostic staging. [file 12916_2019_1445_MOESM1_ESM.pdf]

Additional file 1. Summary of details of studies aimed at evaluating the American Joint Committee on Cancer (AJCC) 8th edition breast cancer prognostic staging.

Study	Cohort	Years	N	Discordance prognostic vs anatomic stage*	HER2+ N	HER2+ treated with trastuzumab (%)	HER2+: Discordance prognostic vs anatomic stage*	HER2+: performance of prognostic stage
Weiss A, JAMA Oncol 2017[1]	MD Anderson (MD) & California Cancer Registry (CCR) Retrospective	2007-2013 (MD) 2005-2009 (CCR)	3327 (MD) 54727 (CCR)	57.6% (MD), 51.6% (CCR) Down: 29.5% (MD), 31.0% (CCR) Up: 28.1% (MD), 20.6% (CCR)	306 (MD) 9676 (CCR)	Not reported	Not reported	Not reported
Plichta J, Annals Surg 2018[2]	National Cancer Database Retrospective**	2010-2014	493854	36.6% Down: 29.7% Up: 6.8%	60155	Not reported	29.4% Down: 29.4% Up: 0%	Not reported
Abdel-Rahman O, Breast Cancer Res Treat 2018[3]	SEER-18 registry Retrospective**	2010-2014	209304	Not reported	25347	Not reported	Not reported	Not reported
Shao N, Cancer Manag Res 2019[4]	SEER-18 registry Retrospective**	2010-2014	168076	53.2% Down: 22.1% Up: 31.1%	25446	Not reported	Not reported	Not reported
Kim I, J Breast Cancer 2018[5]	Korean Breast Cancer Society Retrospective	2009-2012	49248	45.8% Down: 26.1% Up: 19.4%	4964	N=4342 received target therapy	Not reported	Not reported
Liu Y-Y, Future Oncol 2019[6]	SEER database Retrospective**	2010-2014	19608 all triple negative	Not reported in detail	0	-	-	-
Wang M, The Breast 2018[7]	SEER-18 registry Retrospective**	2010-2013	10053 all locally advanced	74% Down: 33% Up: (41%)	2576	Not reported	69% Down: 35.1% Up: 33.9%	Not reported
Lee SB, Breast Cancer Res Treat 2018[8]	Asan Medical Center Retrospective	1999-2008	7458	Not reported	1982	0%	Not reported	Not reported
Wong RX, The Breast 2018[9]	SingHealth institutions Retrospective	2006-2014	6287	46.5% Down: 40.7% Up: 5.8%	1497	86% of 1116 HER2+ patients treated with chemotherapy	Not reported	Not reported

Kurundkar A, Clin Breast Cancer 2018[10]	The University of Alabama at Birmingham Retrospective	1998-2013	6050	52.4%<br>Down: 24.7%<br>Up: 27.7%	561	Not reported	Not reported	Not reported
Kim J-Y, Breast Cancer Res Treat 2018[11]	Samsung Medical Center Retrospective	2004-2008	2790	58.1%<br>Down: 23.4%<br>Up: 34.7%	562	Mostly untreated with trastuzumab	Not reported	DSS log-rank p 0.014 (HR+/HER2+, n=260) and p<0.001 (HR-/HER2+).
Savage P, Eur J Surg Oncol 2019[12]	McGill University Health Centre Retrospective	2010-2013	1703	46.2%<br>Down: 7.5%<br>Up: 38.8%	259	Not reported	Not reported	Not reported
Hu H, World J Oncol 2017[13]	Peking University Shenzen Hospital Retrospective	2011-2016	784	45.7%<br>Down: 27.9%<br>Up: 17.7%	238	Not reported	Not reported	Not reported
Jang N, Virchows Archiv 2019[14]	Yeungnam University Hospital Retrospective	2005-2007	714	45.5%<br>Down: 35.6%<br>Up: 9.9%	129	0%	30.2%<br>Down: 30.2%<br>Up: 0%	Not reported
Ye J, Chin J Cancer Res 2017[15]	Peking University First Hospital Retrospective	2008-2014	421<br>all Luminal A	41.6%<br>Down: 40.2%<br>Up: 1.4%	0	-	-	-
Zhou B, Anticancer Res 2017[16]	Peking University First Hospital Retrospective	2008-2014	170<br>all HER2 enriched	-	170	44%	68.8%<br>All upstaged except 1 patient	OS log-rank p<0.001 for anatomic and prognostic stage

Abbreviations: N, number; OS, overall survival; DSS, disease specific-survival; HR, hormone receptor

*Down: assigned to a more favorable stage by prognostic stage vs anatomic stage (i.e. prognostic stage IA and anatomic stage IB)
Up: assigned to a less favorable stage by prognostic stage vs anatomic stage (i.e. prognostic stage IIA and anatomic stage IB)
**Studies using the National Cancer Database or the SEER registry over a period of time including years 2010-2011. These studies used data partially overlapping with those used by the AJCC panel to develop the prognostic stage.

References

1. Weiss A, Chavez-MacGregor M, Lichtensztajn DY, et al. Validation Study of the American Joint Committee on Cancer Eighth Edition Prognostic Stage Compared With the Anatomic Stage in Breast Cancer. *JAMA Oncol*. 2018;4(2):203-209.

2. Plichta JK, Ren Y, Thomas SM, et al. Implications for Breast Cancer Restaging Based on the 8th Edition AJCC Staging Manual. *Ann Surg*. 2018.

3. Abdel-Rahman O. Validation of the 8th AJCC prognostic staging system for breast cancer in a population-based setting. *Breast Cancer Res Treat*. 2018;168(1):269-275.

4. Shao N, Xie C, Shi Y, et al. Comparison of the 7th and 8th edition of American Joint Committee on Cancer (AJCC) staging systems for breast cancer patients: a Surveillance, Epidemiology and End Results (SEER) Analysis. *Cancer Manag Res*. 2019;11:1433-1442.

5. Kim I, Choi HJ, Ryu JM, et al. Prognostic Validation of the American Joint Committee on Cancer 8th Staging System in 24,014 Korean Patients with Breast Cancer. *J Breast Cancer*. 2018;21(2):173-181.

6. Liu YY, Yu TJ, Liu GY. The predictive value of the prognostic staging system in the 8th edition of the American Joint Committee on Cancer for triple-negative breast cancer: a SEER population-based analysis. *Future Oncol*. 2019;15(4):391-400.

7. Wang M, Chen H, Wu K, Ding A, Zhang M, Zhang P. Evaluation of the prognostic stage in the 8th edition of the American Joint Committee on Cancer in locally advanced breast cancer: An analysis based on SEER 18 database. *Breast*. 2018;37:56-63.

8. Lee SB, Sohn G, Kim J, et al. A retrospective prognostic evaluation analysis using the 8th edition of the American Joint Committee on Cancer staging system for breast cancer. *Breast Cancer Res Treat*. 2018;169(2):257-266.

9. Wong RX, Wong FY, Lim J, Lian WX, Yap YS. Validation of the AJCC 8th prognostic system for breast cancer in an Asian healthcare setting. *Breast*. 2018;40:38-44.

10. Kurundkar A, Gao X, Zhang K, Britt JP, Siegal GP, Wei S. Comparison of AJCC Anatomic and Clinical Prognostic Stage Groups in Breast Cancer: Analysis of 3322 Cases From a Single Institution. *Clin Breast Cancer*. 2018;18(6):e1347-e1352.

11. Kim JY, Lim JE, Jung HH, et al. Validation of the new AJCC eighth edition of the TNM classification for breast cancer with a single-center breast cancer cohort. *Breast Cancer Res Treat*. 2018;171(3):737-745.

12. Savage P, Yu N, Dumitra S, Meterissian S. The effect of the American Joint Committee on Cancer eighth edition on breast cancer staging and prognostication. *Eur J Surg Oncol*. 2019.

13. Hu H, Wei W, Yi X, Xin L, Liu Y. A Retrospective Analysis of Clinical Utility of AJCC 8th Edition Cancer Staging System for Breast Cancer. *World J Oncol*. 2017;8(3):71-75.

14. Jang N, Choi JE, Kang SH, Bae YK. Validation of the pathological prognostic staging system proposed in the revised eighth edition of the AJCC staging manual in different molecular subtypes of breast cancer. *Virchows Arch*. 2019;474(2):193-200.

15. Ye J, Wang W, Xu L, et al. A retrospective prognostic evaluation analysis using the 8th edition of American Joint Committee on Cancer (AJCC) cancer staging system for luminal A breast cancer. *Chin J Cancer Res*. 2017;29(4):351-360.

16. Zhou B, Xu L, Ye J, Xin L, Duan X, Liu Y. The Prognostic Value of the 8th Edition of the American Joint Committee on Cancer (AJCC) Staging System in HER2-Enriched Subtype Breast Cancer, a Retrospective Analysis. *Anticancer Res*. 2017;37(8):4615-4621.
